# Literary Notes

**Published:** 1898-05

**Authors:** 


### LITERARY NOTES.

THE BUFFALO MEDICAL JOURNAL presents itself in the present issue in an entirely new dress, the type for which was specially manufactured for it and will be used exclusively in printing its future editions. At the suggestion of the Buffalo post office we request our exchanges to address us as follows: BUFFALO MEDICAL JOURNAL, 284 Franklin street, Buffalo, N. Y.

POLK'S *Medical and Surgical Directory*, of the United States and Canada, is about to issue a new edition. Physicians who have not already furnished their names should send them at once to R. L. Polk & Company, Detroit, Mich.

THE *International Journal of Surgery* has removed to the Woodbridge Building, 100 William street, New York, where all exchanges should be sent.

THE *Iris* is the title of a book published by the students of the University of Buffalo, to be issued annually, and of which the present number is called volume I. It is a quarto volume of about three hundred pages, printed on heavy plate paper, and quite elaborately illustrated with half-tones and etchings.

The four departments, medicine, law, pharmacy and dentistry, are represented in the work, and it is edited by a committee chosen from these departments, consisting of a board of editors and associate editors. The board of editors is composed as follows: Homer J. Knickerbocker, president; John Livermore Hazen, editor-in-chief; Walter W. Palmer, assistant editor-in-chief; Clifford R. Orr, business manager, and Edward A. Southall, artist. Associate editors: From the department of medicine, William T. Owens, George H. Davis; from the department of pharmacy, Mark H. Minar, Maurice M. Kinsey; from the department of law, John Farrell Koine, Nelson J. Palmer; from the department of dentistry, Miles M. Smith, Frank J. Biker, Nicholas C. Powers; Augustus G. Pohlman, assistant business manager; Albert B. Heist, artist from the dental department.

It is a handsome volume, a credit to all concerned in its publication, and is marred only by the fact that advertisements have been allowed a place between its covers. The presswork and engraving is by the well-known house of Matthews-Northrup Company of Buffalo, which means that it could not have been done better.

---

Messrs. Lea Brothers & Co. announce for early publication the following books: A manual of otology, by Gorham Bacon, A. M., M. D., professor of otology in University Medical College, New York, with an introductory chapter by Clarence J. Blake, M. D., professor of otology in the Harvard Medical school. The treatment of surgical patients before and after operation, by Samuel M. Brickner, M. D., visiting surgeon at the Mt. Sinai Hospital, New York. A text-book of dental pathology, therapeutics and pharmacology, being a treatise on the principles and practice of dental medicine, by Henry H. Burchard, M. D., D. D. S., special lecturer on dental pathology and therapeutics at the Philadelphia Dental College. The principles of treatment, by J. Mitchell Bruce, M. D., F. R. C. P., physician and lecturer on materia medica and therapeutics at Charing-Cross Hospital, London. Diseases of the nose, throat, naso-pharynx and trachea; a manual for students and practitioners, by Cornelius G. Coakley, M. D., professor of laryngology in the University Medical College, New York. Diseases of women; a manual of nonsurgical gynecology, designed especially for the use of students and general practitioners, by Francis H. Davenport, M. D., instructor in gynecology in the medical department of Harvard University. A treatise of gynecology, by E. C. Dudley, A. M., M. D., professor of gynecology in the Chicago Medical College. A text-book of anatomy, by American authors, edited by Frederic Henry Gerrish, M. D., professor of anatomy in the Medical School of Maine. Manual of skin diseases, with special reference to diagnosis and treatment, for the use of students and general practitioners, by W. A. Hardaway, M. D., professor of skin diseases in the Missouri Medical College. Second edition. The principles and practice of obstetrics, by American authors, edited by Charles Jewett, M. D., professor of obstetrics in the Long Island College Hospital, Brooklyn.

---

ARSENAURO and Mercauro is the title of a well-arranged pamphlet issued by the Charles Roome Parmele Company, of New York. It contains the clinical records as reported in the medical journals, resultant from the administration of these two remedies. They are supplied to the profession only through reputable druggists and are made after formulæ furnished by Dr. W. F. Barclay, of Pittsburg. These pamphlets will be supplied upon application to the publishers.

